# Subset of the periodontal ligament expressed leptin receptor contributes to part of hard tissue-forming cells

**DOI:** 10.1038/s41598-023-30446-w

**Published:** 2023-03-01

**Authors:** Hirotsugu Oka, Shinichirou Ito, Mana Kawakami, Hodaka Sasaki, Shinichi Abe, Satoru Matsunaga, Sumiharu Morita, Taku Noguchi, Norio Kasahara, Akihide Tokuyama, Masataka Kasahara, Akira Katakura, Yasutomo Yajima, Toshihide Mizoguchi

**Affiliations:** 1 Department of Oral and Maxillofacial Implantology, Tokyo Dental College, Tokyo, 101-0061 Japan; 2 Department of Pharmacology, Tokyo Dental College, Tokyo, 101-0061 Japan; 3 Department of Oral Pathobiological Science and Surgery, Tokyo Dental College, Tokyo, 101-0061 Japan; 4 Department of Anatomy, Tokyo Dental College, Tokyo, 101-0061 Japan; 5 Oral Health Science Center, Tokyo Dental College, Tokyo, 101-0061 Japan; 6 Tokyo Dental College Research Branding Project, Tokyo Dental College, Tokyo, 101-0061 Japan; 7 Department of Histology and Developmental Biology, Tokyo Dental College, Tokyo, 101-0061 Japan; 8 MDU Hospital, Implant Center, Matsumoto Dental University, Nagano, 399-0781 Japan

**Keywords:** Cell biology, Cellular imaging, Developmental biology, Bone development, Differentiation, Pluripotency, Self-renewal, Stem cells, Stem cells, Adult stem cells, Mesenchymal stem cells, Self-renewal, Stem-cell differentiation

## Abstract

The lineage of periodontal ligament (PDL) stem cells contributes to alveolar bone (AB) and cementum formation, which are essential for tooth-jawbone attachment. Leptin receptor (LepR), a skeletal stem cell marker, is expressed in PDL; however, the stem cell capacity of LepR^+^ PDL cells remains unclear. We used a Cre/LoxP-based approach and detected LepR-cre-labeled cells in the perivascular around the root apex; their number increased with age. In the juvenile stage, LepR^+^ PDL cells differentiated into AB-embedded osteocytes rather than cementocytes, but their contribution to both increased with age. The frequency of LepR^+^ PDL cell-derived lineages in hard tissue was < 20% per total cells at 1-year-old. Similarly, LepR^+^ PDL cells differentiated into osteocytes following tooth extraction, but their frequency was < 9%. Additionally, both LepR^+^ and LepR^−^ PDL cells demonstrated spheroid-forming capacity, which is an indicator of self-renewal. These results indicate that both LepR^+^ and LepR^−^ PDL populations contributed to hard tissue formation. LepR^−^ PDL cells increased the expression of LepR during spheroid formation, suggesting that the LepR^−^ PDL cells may hierarchically sit upstream of LepR^+^ PDL cells. Collectively, the origin of hard tissue-forming cells in the PDL is heterogeneous, some of which express LepR.

## Introduction

The periodontal ligament (PDL) is a connective tissue that sustains attachment of the tooth to the jawbone by penetrating the alveolar bone (AB) and cementum^1^. The PDL is composed of a heterogeneous cell population, including fibroblasts and hard tissue-forming cells, such as osteoblasts and cementoblasts. In addition, it has been postulated that PDL contains a precursor population that generates PDL constituent cells during steady state and regeneration and maintains homeostasis of PDL itself and hard tissue adjacent to PDL throughout life^2–5^. Seo et al.^6^ first defined a human PDL-derived stem cell population positive for STRO-1, a suggested marker of human skeletal stem cells (SSCs)^7^. These PDL stem cell populations have self-renewal and trilineage differentiation potential in vitro and generate cementum-, bone-, and PDL-fibroblast-like structures in vivo post transplantation^6,8–11^. However, where and how PDL stem cells differentiate into descendant cells in vivo or in response to tissue injury remain inconclusive.

Advances in Cre/LoxP-based genetically modified mouse technology have made it possible to track tissue-specific cell populations^12^, leading to the identification and better understanding of the roles and behavior of SSC populations in bone marrow (BM) tissue^13,14^. Using these techniques, PDL stem cells have been explored in vivo and identified as PDL subpopulations that express specific markers such as alpha-smooth muscle actin (αSMA)^15^, Axin2^16^, Gli-1^17,18^, and CD90^19^. Roguljic et al.^15^ reported that αSMA^+^ cells localized in the periapical region of the PDL as perivascular cells and differentiated into PDL fibroblasts, osteoblasts, cementoblasts, and cementocytes in a healthy state and post injury. Meanwhile, Wnt-responsive Axin2^+^ PDL cells differentiate into osteoblasts that form bone tissue in response to tooth extraction^16^ or orthodontic tooth movement^20^, as well as cementoblasts that produce cementum throughout life^19,21^. In addition, Gli1^+^ cells surrounding the neurovascular bundle (NVB) in the apical PDL also suggest the origin of PDL component cells, and their differentiation is positively regulated by the canonical Wnt signaling pathway^17^. Interestingly, Zhao et al.^19^ suggested that during postnatal root formation, both CD90^+^ perivascular cells and Axin2^+^ perivascular-associated cells give rise to cementoblasts; however, in adult tissues, their source is limited to Axin2^+^ cells. In contrast, in periodontal disease, the origin of cementoblasts shifts from Axin2^+^ cells to CD90^+^ cells. Taken together, these studies suggest that the PDL contains a diverse origin of hard tissue-forming cells, each of which may contribute to the maintenance of dental tissue at the same or different appropriate time points; however, the details of the subpopulation of PDL stem cells remain unclear.

It has been suggested that bone homeostasis is regulated by generating osteoblasts in bone developmental and remodeling phases or in regenerative injured bone tissue^14^. Previous lineage-tracing approaches have shown that SSCs can be detected as leptin receptor (LepR)^+^ cells using genetically modified mice that have *LepR-cre* and *floxed-stopped* reporter genes^22,23^. *LepR-cre*-derived osteoblasts are rarely observed in juvenile bone tissues, but osteoblastic differentiation progressively increases with age and eventually becomes the main source of bone tissue. In addition, LepR^+^ cells have been detected in AB, suggesting that they contribute to regenerative bone tissue in extracted sockets^24^. Moreover, LepR^+^ cells localize to the PDL and are a lineage population of Gli1^+^ stem cells in the PDL^17,24^. However, the roles of LepR^+^ PDL cells in the formation of hard tissues, such as AB and cementum, remain unclear.

Therefore, to understand the role of LepR^+^ PDL cells in hard tissue homeostasis in dental tissues, we examined Cre/LoxP-mediated LepR-cre-labeled populations and found that LepR^+^ PDL cells contribute to some of the hard tissue formation in steady state and during regeneration.

## Results

### Hard tissue contribution of LepR-cre-derived PDL cells at the juvenile stage is observed in osteocytes, but not in cementocytes

**Figure 1:**
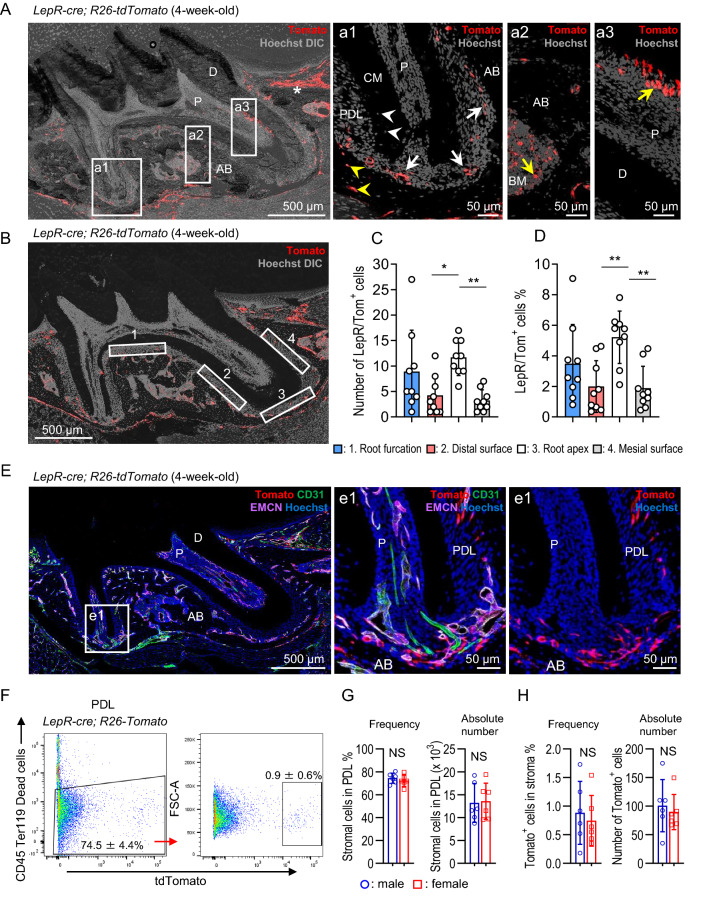
LepR/Tom^+^ PDL cells scarcely differentiate into hard tissue-forming cells in juvenile stages. (**A–E**) Histological analysis of maxillary first molar of 4-week-old *LepR-cre*; *R26-tdTomato* mice. (**A**) Representative confocal images (Z stack) in thick sections; n = 9; scale bar = 500 μm. Numbered panels represent the magnified views of the boxed areas; scale bar = 50 μm. Asterisks: LepR/Tom^+^ cells in gingival tissue, white arrows: LepR/Tom^+^ cells in PDL, yellow arrows: LepR/Tom^+^ cells in bone marrow space or dental pulp, white arrowheads: LepR/Tom^−^ cementocytes, yellow arrowheads: LepR/Tom^+^ osteocytes. Nuclei were visualized using Hoechst. (**B–D**) Quantification of LepR/Tom^+^ cell localization in PDL. Representative image of boxed areas (0.05 mm^2^) used for quantification of LepR/Tom^+^ cells in PDL. 1: Root furcation, 2: Distal surface, 3: Root apex, 4: Mesial surface (**B**). Nuclei were visualized using Hoechst. Absolute number (**C**) and frequency (**D**) of LepR/Tom^+^ cells at indicated area in (**B**). (**C**, One-way ANOVA followed by Tukey’s test) and (**D**, One-way ANOVA followed by Kruskal–Wallis test); n = 9. *p < 0.05, **p < 0.01. Data are represented as mean ± SD. (**E**) Representative confocal images (Z stack) in thick sections stained with anti-EMCN and -CD31 antibodies; n = 3; scale bar = 500 μm. Numbered panels represent the magnified views of the boxed area; scale bar = 50 μm. Nuclei were visualized using Hoechst. (**F–H)** FACS analysis of LepR/Tom^+^ cells in PDL from 4-week-old *LepR-cre*; *R26-tdTomato* mice. (**F**) Representative FACS plots showing the frequency of CD45^−^Ter119^−^ cells (left panel) and LepR/Tom^+^ cells in the stromal PDL population (right panel); n = 6. Quantification of the frequency and absolute number of the CD45^−^Ter119^−^ stromal population in PDL (**G**, Two-tailed Student’s *t*-test) and the LepR/Tom^+^ cells in the CD45^−^Ter119^−^ stromal PDL population (**H**, frequency: Two-tailed Student’s* t*-test; absolute number: two-tailed Mann–Whitney *U*-test); n = 6. NS: not significant. Data are represented as mean ± standard deviation (SD). P: pulp, D: dentin, AB: alveolar bone, PDL: periodontal ligament, CM: cementum, BM: bone marrow, DIC: differential interference contrast, EMCN: endomucin.

Since LepR-expressing cells have been reported in PDL^17,24^, we generated *LepR-cre*; *ROSA26-loxP-stop-loxP-tdTomato* (*R26-tdTomato*) mice to detect LepR-cre-labeled cells in the PDL. In this mouse model, Cre recombinase is expressed in the LepR^+^ cells, and subsequently, the tdTomato stop codon is removed, thereby leading to its expression. Therefore, tdTomato expression in LepR^+^ cells will persist throughout life even after the decrease in LepR levels with cell differentiation. Notably, LepR‐cre is a non-inducible Cre mouse that constitutively expresses Cre in LepR^+^ cells. The LepR-cre‐marked Tomato^+^ population contains both LepR‐cre^+^ cells and their progeny. Confocal microscopic analysis showed that LepR-cre-derived Tomato^+^ cells (LepR/Tom^+^ cells) were present in the PDL of 4-week-old juvenile *LepR-cre*; *R26-tdTomato* mice (Fig. 1A, white arrows). In addition, LepR/Tom^+^ cells were observed in the gingival tissue (Fig. 1A, asterisks), marrow cavity in AB, and dental pulp near the dentin (Fig. 1A, yellow arrows). In contrast, no Tomato^+^ cells were observed around the dental tissue of *R26-tdTomato* control mice (Fig. S1), indicating that Tomato expression is dependent on *LepR-cre* expression. LepR/Tom^+^ cells in the PDL were more abundant in the apical area, and their absolute number and frequency in the root apex were significantly higher than those in both the distal and mesial surfaces of the root (Fig. 1B–D). Similarly, the LepR/Tom^+^ cells in the root furcation were lower than those in the root apex; however, there was no significant difference (Fig. 1B–D). The periapical LepR/Tom^+^ PDL cells were localized adjacent to the blood vessels, as detected via immunostaining for CD31 and endomucin (EMCN) (Fig. 1E). We collected PDL cells from extracted-maxillary molars of *LepR-cre*; *R26-tdTomato* mice and quantified LepR/Tom^+^ cells in the PDL stroma. Flow cytometric analysis showed that the frequency of LepR/Tom^+^ cells was 0.9 ± 0.6% in the PDL stroma, a CD45^−^Ter119^−^ nonhematopoietic population (Fig. 1F, right panel). The frequency and absolute number of stromal cells in the PDL were comparable between the male and female mice (Fig. 1G). Similarly, there was no sex difference in both the frequency and absolute number of LepR/Tom^+^ cells in the stromal PDL (Fig. 1H).

Next, we focused on the contribution of LepR/Tom^+^ cells to osteocytes in AB and found that some were detected as Tomato^+^ cells in 4-week-old mice (Fig. 1A, yellow arrowheads: LepR/Tom^+^ osteocytes). Importantly, immunostaining analysis showed that in 10-month-old mice, LepR expression was observed in the PDL but not in osteocytes, which is consistent with previous studies wherein hard tissue-forming cells were observed to be negative for LepR^22,23,25^ (Fig. S2A, white arrows: LepR^+^ PDL cells; yellow arrows: LepR^−^ osteocytes). Therefore, Tomato^+^ osteocytes might have differentiated from the LepR/Tom^+^ precursor population.

**Figure 2:**
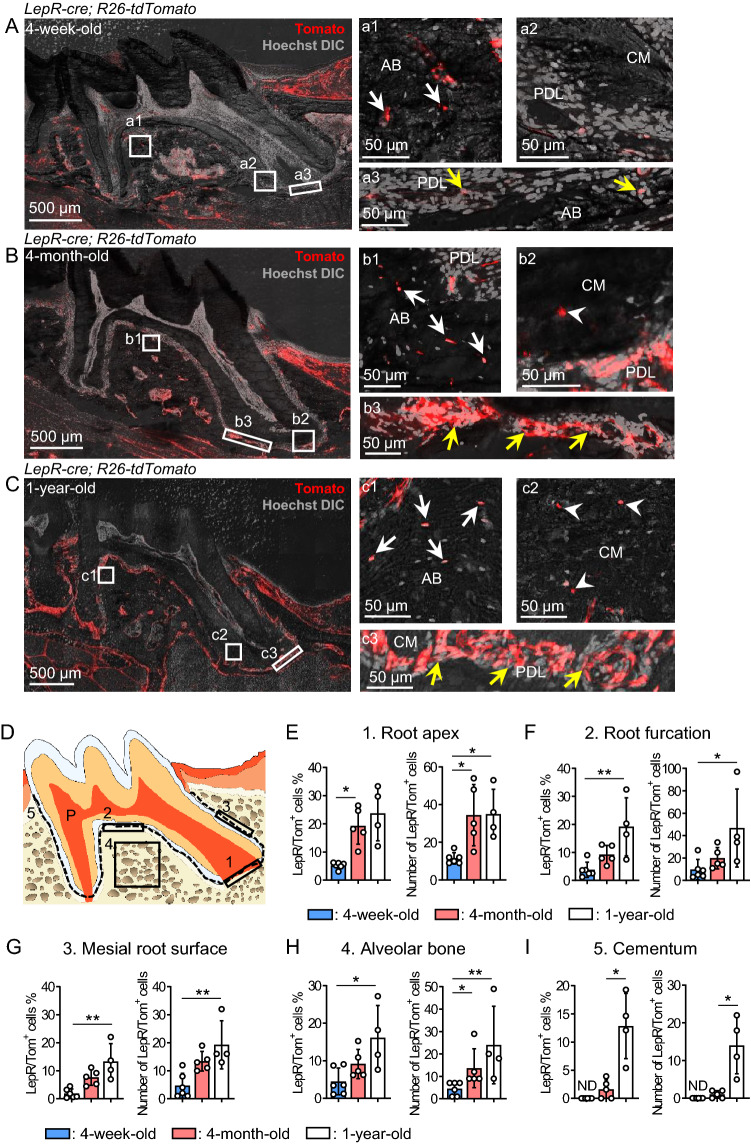
LepR/Tom^+^ PDL cell subpopulation expands and contributes to the AB and cementum during aging. **(A–I)** Time-course analysis of LepR/Tom^+^ cells around dental tissue. (**A–C**) Representative confocal images (Z stack) in thick maxillary first molar sections of 4-week- (**A**, n = 6), 4-month- (**B**, n = 5), and 1-year-old (**C**, n = 4) *LepR-cre*; *R26-tdTomato* mice; scale bar = 500 μm. Numbered panels represent the magnified views of the boxed areas; scale bar = 50 μm. White arrows: LepR/Tom^+^ osteocytes, White arrowheads: LepR/Tom^+^ cementcytes, yellow arrows: LepR/Tom^+^ PDL cells. P: pulp, D: dentin, AB: alveolar bone, PDL: periodontal ligament, CM: cementum, BM: bone marrow, DIC: differential interference contrast. Nuclei were visualized using Hoechst. (**D–I**) Quantification of LepR/Tom^+^ cells around dental tissue. (**D**) Quantification areas in dental tissue. 1: Root apex (0.05 mm^2^), 2: Root furcation (0.05 mm^2^), 3: Mesial root surface (0.05 mm^2^), 4: Alveolar bone (0.25 mm^2^), 5: The dotted line, the cementum between the mesial and distal cemento-enamel junction. (**E–I**) Frequency and absolute number of LepR/Tom^+^ cells in the areas as shown in D at the indicated time point. (**E**) Root apex (left panel: Welch’s one-way ANOVA; right panel: One-way ANOVA followed by Tukey’s test). (**F**) Root furcation (left panel: One-way ANOVA followed by Tukey’s test; right panel: One-way ANOVA followed by Kruskal–Wallis test). (**G**) Mesial root surface (One-way ANOVA followed by Tukey’s test). (**H**) Alveolar bone (left panel: One-way ANOVA followed by Tukey’s test; right panel: One-way ANOVA followed by Kruskal–Wallis test). (**I**) Cementum (Two-tailed Welch’s *t*-test between 4-week- and 4-month-old mice); n = 4–6. *p < 0.05, **p < 0.01. NS: not significant. Data are represented as mean ± standard deviation (SD).

However, in agreement with long bone tissue reports that LepR^+^ SCCs contribute minimally to neonatal hard tissue formation^22,23^, the frequency of LepR/Tom^+^ PDL-derived osteocytes per total was only 4.53 ± 3.56% in 4-week-old mice (0.25 mm^2^/AB, n = 6) (Fig. 2H, left panel).

Furthermore, all cementocytes were negative for LepR/Tom in 4-week-old mice (Fig. 1A, white arrowheads: LepR/Tom^−^ cementocytes and Fig. 2I). These results indicate that LepR/Tom^+^ cells are localized in the PDL, but do not function as the main source of hard tissue forming cells, osteocytes, and cementocytes during juvenile stages.

### LepR/Tom^+^ PDL cells are expanded in PDL tissues and differentiate into both osteocytes and cementocytes during aging

We next performed time-course analyses of LepR/Tom^+^ PDL cells for up to 1 year to determine their contribution to osteocytes and cementocytes throughout their lifetime. Whole dental tissue imaging showed that the levels of LepR/Tom^+^ cells were increased in PDL, AB, and cementum in 1-year-old mice, but remained unchanged in gingival and dental pulp (Fig. 2A–C). We quantified the frequency and absolute number of LepR/Tom^+^ cells localized in each specific area, as shown in Fig. 2D, and found that LepR/Tom^+^ PDL cells in the apical region significantly increased from four weeks to four months of age (Fig. 2E). Similarly, both the frequency and absolute number of LepR/Tom^+^ PDL cells localized in the furcation or mesial root surface of 1-year-old mice were significantly higher than those of 4-week-old mice (Fig. 2F, G). We further quantified LepR/Tom^+^ cells embedded in the hard tissue, as indicated in Figs. 2D, 4, and 5, and found that the contribution of LepR/Tom^+^ PDL cells to osteocytes and cementocytes significantly increased with age (Fig. 2H, I). Since we did lineage tracing analyses in up to 1-year old animals and found age-dependent differences in several parameters, we selected 10-month-old mice to evaluate LepR expression in distinct cell types. Immunostaining for LepR showed that the cementocytes and osteocytes in 10-month-old mice were negative for LepR; therefore, it can be considered that Tomato^+^ cementocytes and osteocytes were differentiated from LepR/Tom^+^ PDL cells (Fig. S2A; yellow arrowheads: LepR^−^ cementocytes; yellow arrows: LepR^−^ osteocytes).

However, the frequency of LepR/Tom^+^ cells among hard tissue-embedded cells remained lower than 20%, even in 1-year-old mice (16.17 ± 8.56% of osteocytes, 12.85 ± 5.81% of cementocytes; Fig. 2H, I). Altogether, these results suggest that LepR^+^ PDL cells are sustained for more than 1 year, and some differentiate into part of the hard tissue-forming cells, such as osteocytes and cementocytes, albeit at a low frequency.

### LepR/Tom^+^ PDL cells contribute to part of the osteocytes in regenerated bone tissue of tooth extraction sockets

**Figure 3:**
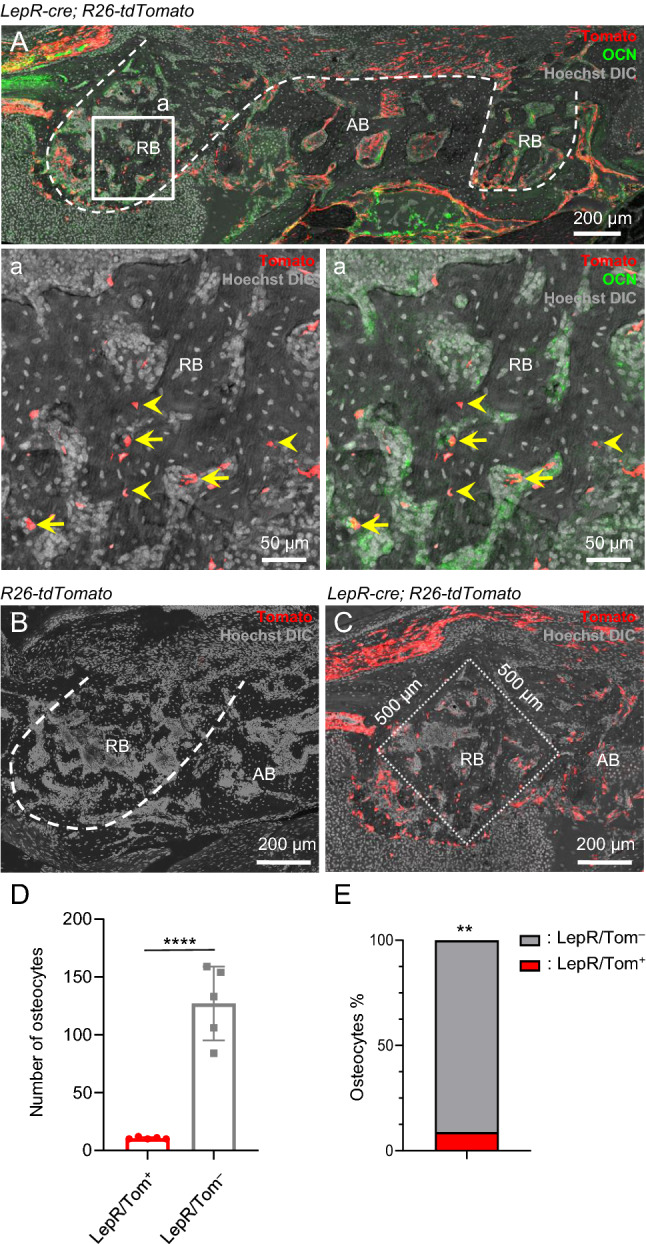
LepR/Tom^+^ PDL cells contribute as some of the osteocytes in regenerative bone tissue of the extraction socket. **(A–E)** Lineage tracing analysis of LepR/Tom^+^ cells in regenerative bone (RB) tissue of extraction socket. **(A, B)** Representative confocal images (Z stack) in thick sections of regenerated bone in the socket of maxillary first molars after 2 weeks post tooth extraction in 4-week-old *LepR-cre*; *R26-tdTomato* mice stained with anti-OCN antibody (**A**, n = 5) or in 4-week-old *R26-tdTomato* control mice (**B**, n = 3). Scale bar = 200 μm. Lower panels represent the magnified views of the boxed areas; scale bar = 50 μm. Arrows: LepR/Tom^+^OCN^+^ osteoblasts, arrowheads: LepR/Tom^+^ osteocytes. Dotted lines indicate the border of the extraction socket. Nuclei were visualized using Hoechst. (**C–E**) Quantification of LepR/Tom^+^ and LepR/Tom^−^ osteocytes in RB tissue of the extraction socket. (**C**) Representative quantification area in RB (0.25 μm^2^). Scale bar = 200 μm. Nuclei were visualized using Hoechst. (**D**) Absolute number of osteocytes in RB (Two-tailed Student’s *t*-test). (**E**) Frequency of LepR/Tom^+^ and LepR/Tom^−^ osteocytes in RB (two-tailed Mann–Whitney *U*-test); n = 5. **p < 0.01, ****p < 0.0001. Data are represented as mean ± standard deviation (SD). OCN: osteocalcin, RB: regenerated bone, AB: alveolar bone, DIC: differential interference contrast.

PDL-derived stem cells have been suggested to differentiate into osteogenic cells and contribute to the regeneration of hard tissue in tooth extraction sockets^16,26^. Therefore, we analyzed the contribution of LepR/Tom^+^ PDL cells to bone-forming cells in regenerative bone (RB). The maxillary first molars of *LepR-cre*; *R26-tdTomato* mice were extracted, and 2 weeks later, the extraction socket that was filled with RB was analyzed. LepR/Tom^+^ cells were observed as osteocalcin (OCN)^+^ osteoblasts and osteocytes in the RB in the mesial root area (Fig. 3A, yellow arrows: LepR/Tom^+^ OCN^+^ osteoblasts; yellow arrowheads: LepR/Tom^+^ osteocytes). Immunostaining for LepR showed that the osteocytes embedded in the RB were negative for LepR; therefore, it can be considered that Tomato^+^ cells were differentiated from LepR/Tom^+^ precursor (Fig. S2B; yellow arrows: LepR^−^ osteocytes). In contrast, no Tomato^+^ cells were found in the regenerative bone tissue of *R26-tdTomato* control mice, indicating that Tomato expression is dependent on *LepR-cre* in regenerative tissue (Fig. 3B). We next quantified the number of LepR/Tom^+^ and LepR/Tom^−^ osteocytes in the region of interest (Fig. 3C) and found that the number of LepR/Tom^+^ osteocytes was significantly lower than that of LepR/Tom^−^ osteocytes in regenerative bone tissues (Fig. 3D). The frequency of LepR/Tom^+^ osteocytes among the total regenerative osteocytes was lower than 9%, suggesting that most of the bone-forming cells in the extraction socket were derived from precursors other than LepR/Tom^+^ (Fig. 3E). These results suggest that LepR/Tom^+^ PDL cells assist in bone tissue regeneration, while the LepR/Tom^−^ PDL subpopulation simultaneously contributes more to bone tissue regeneration in the extraction socket.

### Both LepR/Tom^+^ and LepR/Tom^−^ PDL cell populations express self-renew stem cell activity in vitro

**Figure 4:**
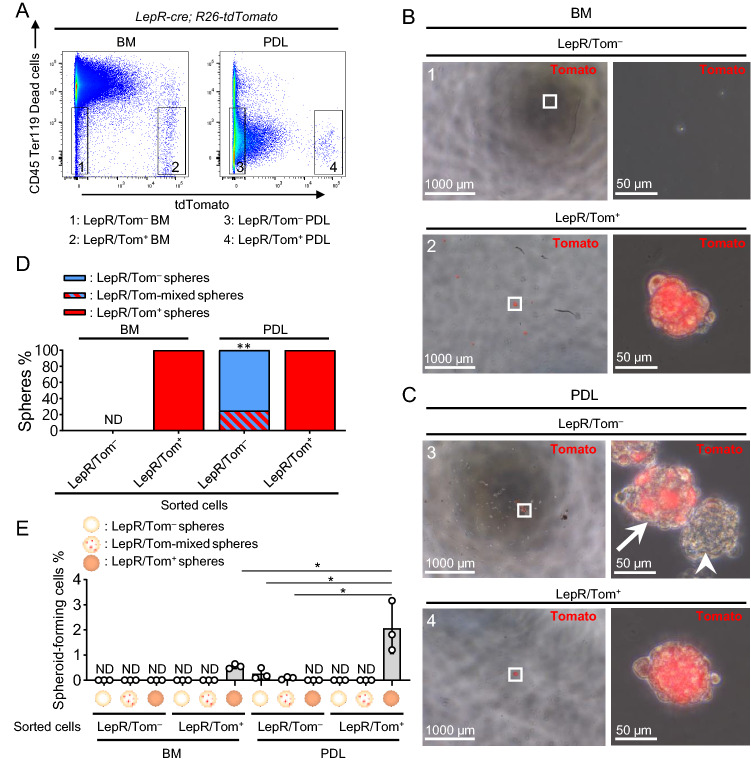
Self-renew stem cell activity is exerted in both LepR/Tom^+^ and LepR/Tom^−^ PDL cell populations.** (A–E)** Sphere formation assay of bone marrow (BM)- and periodontal ligament (PDL)-derived LepR/Tom^+^ or LepR/Tom^−^ stromal populations. (**A**) Representative FACS plots showing sorted population of BM (left panel, 1: LepR/Tom^−^, 2: LepR/Tom^+^) and PDL cells (right panel, 3: LepR/Tom^−^, 4: LepR/Tom^+^) from *LepR-cre*; *R26-tdTomato* mice. (**B,C**) Representative images of spheres derived from BM (**B**) and PDL (**C**). Arrows: LepR/Tom-mixed spheres, arrowheads: LepR/Tom^−^ spheres; scale bar = 1000 μm. Right panels are the magnified views of the boxed areas; scale bar = 50 μm. The panel numbers correspond to the gated number in (**A**). (**D**) Frequency of spheroid types; n = 3. Two-tailed Student’s *t*-test between LepR/Tom^−^ and LepR/Tom-mixed spheres derived from sorted LepR/Tom^−^ PDL cells. **p < 0.01. (**E**) Frequency of sphere forming cells in the sorted cells; n = 3. One-way ANOVA followed by Tukey’s test among the sphere-detected groups. *p < 0.05. Data are represented as mean ± standard deviation (SD). BM: bone marrow, PDL: periodontal ligament.

**Figure 5:**
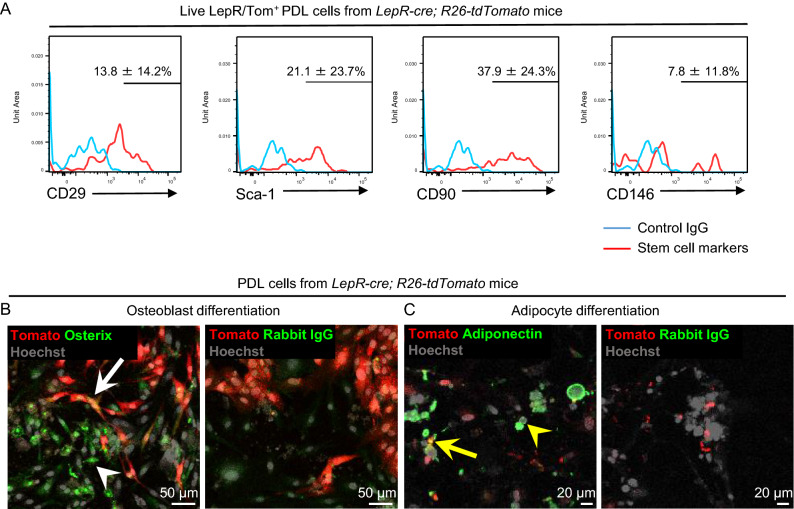
LepR/Tom^+^ PDL cells express stem cell markers, while both LepR/Tom^+^ and LepR/Tom^−^ PDL cells possess in vitro pluripotency.** (A)** Representative FACS plots (gated on live LepR/Tom^+^ cells) showing the frequency of stem cell marker expression in LepR/Tom^+^ PDL cells; n = 3. Blue and red lines represent isotype controls and antibodies against the stem cell markers indicated, respectively. (**B, C**) Pluripotency of LepR/Tom^+^ and LepR/Tom^−^ PDL cells. Representative images of differentiated osteoblasts (**B**) and adipocytes (**C**) derived from PDL cells of *LepR-cre; R26-tdTomato* mice; n = 3. Osteoblasts or adipocytes were detected via staining for osterix (**B**, green) or adiponectin (**C**, green), respectively. Right panels are stained for control rabbit IgG. White arrows: LepR/Tom^+^/osterix^+^ osteoblasts, white arrowheads: LepR/Tom^−^/osterix^+^ osteoblasts, yellow arrows: LepR/Tom^+^/adiponectin^+^ adipocytes, yellow arrowheads: LepR/Tom^−^/adiponectin^+^ adipocytes. Nuclei were visualized using Hoechst; scale bar = 50 μm (**B**) and 20 μm (**C)**.

Our lineage tracing analysis of LepR/Tom^+^ cells suggests that the heterogeneous origin of hard tissue-forming cells is included in the PDL and works in both healthy and injured conditions. To clarify this point, the self-renewal capacity of both LepR/Tom^+^ and LepR/Tom^−^ PDL cell subpopulations was examined by sphere-forming activity under non-adherent culture conditions^27–29^. Since the sphere-forming activity of long bone stroma has been reported to be enriched in the LepR/Tom^+^ stromal population, BM-derived LepR/Tom^+^ stromal cells were used as a positive control for the sphere-forming assay^29^. *LepR-cre*; *R26-tdTomato* mice-derived LepR/Tom^+^ and LepR/Tom^−^ BM cells or PDL stromal subpopulations were collected using a cell sorter, and a sphere formation assay was performed on each subpopulation (Figs. 4A and S3). Consistently, BM-derived sphere-forming activity was observed only in LepR/Tom^+^ BM stroma but not in LepR/Tom^−^ BM stroma (Fig. 4B, D, E). In contrast, PDL-derived LepR/Tom^−^ cells expressed sphere-forming activity, and these spheres were composed of LepR/Tom^−^ cells or LepR/Tom-positive and LepR/Tom-negative complexes (referred to as LepR/Tom-mixed) (Fig. 4C, arrows: LepR/Tom-mixed spheres, arrowheads: LepR/Tom^−^ spheres). The frequency of LepR/Tom-mixed spheres of the total spheres derived from LepR/Tom^−^ PDL cells was significantly lower than that of LepR/Tom^−^ spheres (Fig. 2D). In contrast, LepR/Tom^+^ PDL cells also had sphere-forming activity and were composed only of LepR/Tom^+^ cells (Fig. 4C–E). Remarkably, PDL-derived LepR/Tom^+^ cells had a significantly higher rate of sphere formation in sorted cells than BM-derived LepR/Tom^+^ cells (Fig. 4E). Consistent with these results, part of the LepR/Tom^+^ PDL cells expressed stem cell markers, such as CD29, Sca-1, CD90, and CD146^19,30,31^ (Fig. 5A), while stemness in both LepR/Tom^+^ and LepR/Tom^−^ PDL cells was also suggested by their pluripotency as demonstrated by osteoblast and adipocyte differentiation (Fig. 5B and C). Altogether, these results suggest that in vitro self-renewal stem cell activity in PDL-derived stroma is expressed in a heterogeneous population, both in LepR/Tom^+^ and LepR/Tom^−^ cells. In addition, LepR/Tom^−^ PDL cells have been suggested to give rise to LepR/Tom^+^ PDL cells during in vitro sphere formation.

## Discussion

Over the past few years, lineage-tracking approaches to identify stem cell populations in the PDL have been performed, and several Cre-expressing mouse lines, such as *αSMA-creER*, *Axin2-creER*, and *Gli-1-creER*, have been shown to be useful for the detection of stem cell populations^32^. In this study, we examined the roles of the LepR^+^ population in PDL using *LepR-cre* and *floxed-stopped* reporter mice and found that they expressed stem cell capacity to give rise to osteocytes and cementocytes in healthy and hard tissue regenerative conditions. However, the contribution rate of LepR/Tom^+^ PDL cells to the lineage of hard tissue-forming cells was lower than that of the LepR/Tom^−^ subpopulation. In addition, PDL-derived self-renewal stem cell activity was found in both LepR/Tom^+^ and LepR/Tom^−^ populations in spheroid formation assays using in vitro culture. These results suggest that PDL stem cells may be composed of heterogeneous populations, one of which expresses LepR.

Unfortunately, our genetic lineage tracing analysis was performed using a non-inducible Cre mouse line that constitutively expresses the cre gene in LepR^+^ cells. In this system, since cellular labeling with Tomato fluorescence is constantly induced corresponding to the levels of LepR expression, it is essential to confirm the LepR expression levels in progeny cells, such as the osteocytes and cementocytes that were focused on in our study. Although our immunostaining data indicated negative LepR in hard tissue-forming cells in healthy and regenerative tissue (Fig. S2), further analysis using inducible Cre mice line, *LepR-creERT2*, remains warranted to confirm the hard tissue lineage contribution of LepR^+^ PDL cells^33,34^.

Although *LepR-cre* labeled multiple areas of dental tissue in addition to PDL, we believe that LepR/Tom^+^ PDL cells are the most likely origin of hard tissue-forming cells for the following reasons: First, LepR/Tom^+^ PDL cells are localized closer to the cementum and AB than LepR/Tom^+^ cells in other areas, and second, LepR/Tom^+^ PDL cells increased with age, while the number of LepR/Tom^+^ gingiva and dental pulp cells remained relatively the same throughout life. Further studies, such as lineage-tracing analysis of specifically labeled LepR/Tom^+^ PDL cells, are warranted to further clarify this.

*Gli1-creER*-labeled cells have been suggested to be PDL stem cells located near the apical NVB^17^. Interestingly, LepR/Tom^+^ cells were localized mostly at the root apex, as well as *Gli1-creER*-labeled cells, and were adjacent to blood vessels (Fig. 1B–E). These distribution patterns may suggest a hierarchical relationship between LepR^+^ and Gli1^+^ cells in the PDL. Previous genetic lineage tracing approaches suggest that part of the lineage for Gli1^+^ cells expressed LepR^17^. In addition, since the LepR/Tom^−^ cell population of PDL cells contained self-renewal stem cells, some of which increased LepR expression during sphere formation (Fig. 4C), LepR^+^ cells may sit hierarchically downstream of Gli1^+^ or other stem cell populations in the PDL cells. Alternatively, as previous studies have suggested that the stem cell properties increased during spheroid culture condition^35,36^, one of the stem cell markers, LepR, may be increased in LepR/Tom^−^ population of PDL cells. Consistently, LepR/Tom^+^ cells in the PDL cells also have sphere-forming activity on their own, suggesting that LepR/Tom^+^ cells maintain self-renewal activity (Fig. 4C–E). The frequency of spheroid-forming cells in LepR/Tom^+^ PDL cells was significantly higher than that in LepR/Tom^+^ cells derived from BM (Fig. 4E); hence, the self-renewing cell population was more enriched in LepR/Tom^+^ PDL cells than in LepR/Tom^+^ BM cells. However, the characteristics of spheroid-forming cells in LepR/Tom^+^ PDL cells remain to be determined. Recent single-cell RNA sequencing technology has ushered in a new era for the study of BM stromal populations and revealed that LepR/Tom^+^ BM cells are composed of a heterogeneous cell population, including SSCs and their progeny cells^37^. Future single-cell transcriptomic approaches will provide additional information to characterize the spheroid-forming population in LepR/Tom^+^ PDL cells.

It has been suggested that LepR^+^ BM stromal cells infrequently differentiate into osteoblasts in long bone tissue at the neonatal stage, but their osteoblastic contribution gradually increases with age^22,23^. In addition, a dual-recombinase lineage tracing system revealed that independent progenitor cells, chondrocytes, and LepR^+^ BM stroma gave rise to osteoblasts before and after adolescence^33^. These findings suggest a diverse origin of osteoblasts, which differentiate depending on the in vivo environmental conditions such as rapid longitudinal bone growth and slow bone remodeling. In addition to these observations in growing long bone tissue, a previous study has shown that the frequency of *LepR-cre*-derived lineage per total nucleated cells in AB was only approximately 4.22% in 4-week-old mice, but reached 20.69% at 3 months of age^24^. Although their study focused on the LepR^+^ cells localized in AB but not in the PDL, we and others^24^ revealed that LepR^+^ cells in dental tissue work as the origin of osteocytes in AB, and the contribution was rarely observed at juvenile stages, similar to long bone tissue. Conversely, the frequency of AB osteocytes derived from *Gli1-creER*^+^ cells labeled around 5–8-week-old was only 5% after 30 days of lineage tracing, but their contribution increased up to 85% after 8 months of observation. In contrast, the frequency of LepR/Tom^+^ cells in AB osteocytes was < 17%, even in 1-year-old mice (Fig. 2H). These results indicate that most AB osteocytes are derived from Gli1^+^ cells and a small population from LepR^+^ cells, although more details of the hierarchical relationship between these two populations remain be clarified.

Our in vivo lineage tracing data indicate that although LepR/Tom^+^ PDL cells function as the origin of cementocytes, other PDL cell populations also supply cementocytes. Previous genetic lineage tracing approaches have shown that PDL cell populations labeled with *αSMA-creER*^15^, *Axin2-creER*^21^, or *Gli1-creER*^17,38^ differentiate into cementocytes in vivo. Approximately 60–70% of cementocytes in 8-week-old-mice were derived from Gli1^+^ or Axin2^+^ PDL cells, which are labeled around 3–4 weeks of age^21,38^. Additionally, *keratin 14-cre*-labeled Hertwig’s epithelial root sheath has also been suggested as a source of cementocytes^39^, although the conclusion remains controversial^21^. Importantly, Gli1^+^ PDL cells function as the origin of cementocytes at 4 weeks of age, albeit at a low frequency^38^, when LepR/Tom^+^ PDL cell-derived cementocytes are not observed in the cementum (Fig. 2I). These results suggest that the origin of cementocytes switches from the growth to adult stages. Alternatively, Gil1^+^ PDL cell may give rise to the LepR^+^ lineage, which contributes to cementogenesis after adolescence^17^.

Similar to healthy dental tissue, osteocytes in the regenerated extraction socket were derived from not only LepR^+^ PDL cells but also other populations. Axin2^+^ PDL stem cells contribute to socket healing via differentiation into osteoblasts^16^. In addition to the PDL population, Gli1^+^ stem cells localized in AB were also suggested to provide bone lineage during bone regeneration in the extraction socket, which physically removes the remaining PDL^40^. Importantly, despite the modest contribution of LepR^+^ lineage cells to regenerative bone tissue, a previous study performed using the Cre/LoxP-based cell depletion technique showed that the depletion of LepR^+^ lineage cells significantly diminished bone regeneration in the extraction socket^24^. These findings suggest that the LepR^+^ cell lineage plays an essential role in bone regeneration in addition to providing bone-forming cells.

In summary, our study demonstrated that the hard tissue-forming cells in the dental tissue were derived from diverse origins, one of which was detected as LepR/Tom^+^ PDL cells. However, their characteristics, specific roles in the PDL, and relationships with other PDL stem cell populations remain to be elucidated. In addition, the existence of the LepR^+^ PDL cell population in human tissue must be clarified. Nevertheless, our data highlight the previously unknown lineage of LepR^+^ PDL cells in healthy and damaged dental tissues, which significantly contributes to the hard tissue maintenance in health and dentistry, such as for dental implantation.

## Methods

### Experimental animals

C57BL/6 mice were purchased from Sankyo Labo Service Corporation (Tokyo, Japan). *B6.129(Cg)-Lepr*^*tm2(cre)Rck*^*/J (Lepr-cre),* (JAX008320)^41^, and *B6.Cg-Gt(ROSA)26Sor*^*tm14(CAG-tdTomato)Hze*^*/J*, (*R26-tdTomato*), (JAX007914)^42^ mice were purchased from the Jackson Laboratory (Bar Harbor, ME, USA). All transgenes were used as heterozygotes in the experiments. To generate *LepR-cre*; *R26-tdTomato* heterozygote mice, we crossed *LepR-cre* homozygote male mice with *R26-tdTomato* homozygote female mice. Unless otherwise stated, male mice were used in the experiments. The following number of *LepR-cre*; *R26-tdTomato* heterozygote mice were used in this study: Fig. 1: 18 mice; Fig. 2: 15 mice; Fig. 3: 5 mice; Fig. 4: 3 mice; Fig. 5: 6 mice. During the experiments, euthanasia was performed by cervical spine fracture dislocation under isoflurane anesthesia (Pfizer Inc., New York City, NY, USA). The mice were fed a regular diet (MF; Oriental Yeast Co., Ltd., Tokyo, Japan). The maximum number of mice per cage was five. All mice were maintained under specific pathogen-free conditions at 24 ± 2 °C, exposed to 50–60% humidity with a 12-h light/dark cycle, and provided with sterile water and ad libitum access to food in animal facilities certified by the Animal Care and Use Committees of Tokyo Dental College (Tokyo, Japan). All experimental protocols were approved by Animal Care Committee of the Tokyo Dental College (Tokyo, Japan). (No. DNA1840, 214102, 224102). All methods were carried out in accordance with the guidelines of the Animal Care Committee of Tokyo Dental College (Tokyo, Japan). All methods are reported in accordance with ARRIVE guidelines.

### Antibodies and reagents

The following primary antibodies were used: rat anti-endomuchin antibody (V. 7C7) (Santa Cruz Biotechnology, CA, USA) (1:100), goat anti-mouse leptin receptor (R&D SYSTEMS, Minneapolis, MN, USA) (1:100), goat anti-mouse-CD31antibody coupled to Alexa Fluor (AF) 488 (BD Biosciences, San Jose, CA, USA) (1:50), rat anti-mouse osteocalcin antibody (R21C-01A) (1:200) (Takara, Shiga, Japan), rat anti-CD45 antibody coupled to allophycocyanine (APC) (30-F11) (1:500) and rat anti-Ter119 (TER-119) antibody coupled to APC (1:500) (both from Thermo Fisher Scientific, Waltham, MA, USA), rat anti-Ly-6A/E (Sca-1) antibody coupled to AF 488 (D7) (1:500), hamster anti-CD29 antibody coupled to AF 488 (HMβ1-1) (1:500), rat anti-CD90.2 antibody coupled to AF 488 (30-H12) (1:500), rat anti-CD146 antibody coupled to AF 488 (ME-9F1), isotype control rat IgG2a, κ coupled to AF 488 (RTK2758) (1:500) and isotype control hamster IgG coupled to AF 488 (HTK888) (1:500) (all from BioLegend, San Diego, CA, USA), rabbit anti-adiponectin antibody (1:10) (Novus Biologicals, Centennial, CO, USA), rabbit anti-osterix antibody (1:100) (Abcam, Cambridge, UK) and normal rabbit IgG (60024B) (R&D SYSTEMS).

Donkey anti-rat coupled to AF 647 (1:1000) (Abcam), donkey anti-rabbit coupled to AF 488 (1:1000) and donkey anti-goat coupled to AF 647 (1:1000) (both from Thermo Fisher Scientific) were used as the secondary antibodies for immunostaining. Nuclei were stained using Hoechst 33342 (Thermo Fisher Scientific). Dissection, sectioning, and staining for each experiment were always performed at the same time and conditions.

### Preparation of BM and PDL cell suspension

Mouse BM cells were flushed from the right femora using 1 mL of collagenase/protease mixed buffer (1 Wunsch units of Liberase™ and 0.5 mg/mL Pronase) (both from Roche, Mannheim, Germany) in Hanks’ Balanced Salt Solution (HBSS) with Ca^2+^, Mg^2+^ (Thermo Fisher Scientific)^43^. Mouse BM cells or maxillary molars were incubated with 1 mL of collagenase/protease mixed buffer for 30 min at 37 °C in a shaking incubator (Thermomixer C, Eppendorf, Hamburg, Germany). The digested BM and PDL cells were treated with hemolysis buffer (ACK Lysing Buffer, Thermo Fisher Scientific) for 3 min on ice, washed with phosphate-buffered saline (PBS) containing 2% fetal bovine serum (FBS) (all from Fujifilm Wako Pure Chemical, Osaka, Japan) and 0.5 mM ethylenediaminetetraacetic acid (EDTA, Thermo Fisher Scientific), and used for subsequent experiments.

### Flow cytometric analysis and cell sorting

Cells were stained with antibodies, while flow cytometry or cell sorting experiments were performed using a FACSMelody (BD Biosciences) equipped with FACSChorus software (BD Biosciences). L-15 medium (21083-027) containing, 1% Antibiotic–Antimycotic (100 units/mL penicillin, 100 µg/mL streptomycin, 0.25 µg/mL amphotericin B), 10 mM HEPES (all from Thermo Fisher Scientific), and 1 mg/mL Bovine Serum Albumin (BSA, Fujifilm Wako Pure Chemical) sterilized with 0.02 μm filter was used for cell preparation for cell sorting. Dead cells and debris were excluded using FSC, SSC, and SYTOX™ Red Dead Cell Stain (Thermo Fisher Scientific) staining profiles. Data were analyzed using FlowJo V10 software (BD Biosciences).

### Spheroid formation assay

Cells collected by the cell sorter were cultured in non-adherent 24 well plates (Corning, New York, NY, USA) (LepR/Tom^+^ BM cells: 4781‒9849 cells/well, LepR/Tom^−^ BM cells: 18,642‒201,149 cells/well, LepR/Tom^+^ PDL cells: 83‒348 cells/well, LepR/Tom^−^ PDL cells: 9125‒61,003 cells/well) with spheroid-forming media^22,27–29^ (1:2 ratio of DMEM/F-12 (1:1) (21331-020) and Human Endothelial Medium (11111-044) supplemented with 3.75% Chicken Extract, 0.1 mM β-ME, 1% Non-essential amino acids, 1% Antibiotic–Antimycotic, 1% N2, 2% B27, 20 ng/mL mouse PDGF-AA (all from Thermo Fisher Scientific), 20 ng/mL human bFGF (ReproCELL Inc., Kanagawa, Japan), 20 ng/mL mouse oncostatin M, 20 ng/mL mouse IGF-1 (all from R&D SYSTEMS), 20 ng/mL mouse EGF (Sigma-Aldrich, St. Louis, MO, USA)). After culturing for 14 days, spheroid-forming efficiency was determined. Fluorescence and phase-contrast images of spheroids were acquired using an All-in-One Fluorescence Microscope (BZ-X700) equipped with a BZ-X-Viewer, BZ-X Analyzer (all from KEYENCE, Osaka, Japan), a CFI Plan Fluor DL (4×/0.13), and a CFI Plan Fluor DL (10×/0.45) (both from Nikon, Tokyo, Japan).

### Tooth extraction

The maxillary molars were extracted under anesthesia with triple anesthesia (medetomidine hydrochloride 0.75 mg/kg (Domitol, Nippon Zenyaku Kogyo Co.,Ltd. Fukushima, Japan), midazolam 4.0 mg/kg (Dormicum, Sandoz K. K., Tokyo, Japan), butorphanol 5.0 mg/kg (Vetorphale, Meiji Seika Pharma Co., Ltd. Tokyo, Japan)) using hooked-end forceps. After surgery, mice were injected with Atipamezole 0.75 mg/kg (antisedan, Nippon Zenyaku Kogyo Co.,Ltd.) to antagonize the anesthesia effect. Two weeks later, the tissue from the extraction socket was collected and used for analysis.

### Microscopy and histomorphometry

Mouse maxillae were removed and fixed with 4% paraformaldehyde (PFA) for 24 h at 4 °C. The dissected maxilla was decalcified using 20% Morse solution (Fujifilm Wako Pure Chemical) in sterilized water for 24 h at 4 °C and subsequently incubated with 10, 20, and 30% sucrose solutions for more than 2 h at 4 °C for cryoprotection. The samples were embedded in super-cryo-embedding medium (SCEM; Section-Lab, Hiroshima, Japan). Cryosections, 16-µm-thick, were cut in accordance with Kawamoto’s film method using Cryofilm type 4D (16UF) and a tungsten carbide microtome (Section-Lab)^44^. The sections were incubated with primary antibodies for 24 h at 24 ± 2 °C; however, in case of staining with rat anti-endomuchin antibody and goat anti-mouse-CD31antibody coupled to AF 488, the tissue was pretreated with 0.25% Triton X-100 (Sigma-Aldrich) for 10 min at 24 ± 2 °C. The sections were further incubated with secondary antibodies for 2 h at 24 ± 2 °C, mounted using 30% glycerol, covered with coverslips, and sealed with nail polish. Z stacks of confocal images were obtained at 1-µm intervals between 16-µm-thick sections. Fluorescence images were acquired using a laser-scanning confocal microscope (LSM880) equipped with a Plan-APOCHROMAT (20 × /0.8), ZEN 2.3 black edition, and ZEN 2.6 blue edition (all from Carl Zeiss, Oberkochen, Germany).

### In vitro cell differentiation

PDL cells of *LepR-cre*; *R26-tdTomato* mice were cultured at 37 °C and 5% CO_2_ in 96-well plates (1.5 × 10^5^ cells/well) (Corning) up to subconfluent density with 200 μl DMEM (10567-014) containing 1% Antibiotic–Antimycotic and 10% mesenchymal stem cell-qualified FBS (12662-011) (all from Thermo Fisher Scientific); in case of osteoblastic differentiation, the 96-well plats were coated with 10% Cellmatrix (Type I-A) (Nitta Gelatin Inc., Osaka, Japan). For osteoblastic differentiation, cells were cultured with αMEM (M4526) containing 5 mM β-glycerophosphate, 100 μg/mL ascorbic acid (all from Sigma-Aldrich), 10% FBS (Fujifilm Wako Pure Chemical), 1% Antibiotic–Antimycotic, 2 mM L-Glutamine (both from Thermo Fisher Scientific), and 200 ng/mL BMP2 (R&D SYSTEMS) for 1 week. Medium was changed every 2–3 days. Adipocytic differentiation was induced using AdipoInducer Reagent (for animal cell) (Takara) kit, according to the manufacturer’s instructions. Briefly, cells were cultured with RPMI 1640 medium containing 10% FBS (both from Fujifilm Wako Pure Chemical), 1% Antibiotic–Antimycotic (Thermo Fisher Scientific), insulin (10 μg/mL) and dexamethasone (2.5 μM) at 37 °C and 5% CO_2_ for 48 h; the medium was replaced with RPMI 1640 medium containing 10% FBS, 1% Antibiotic–Antimycotic and insulin (10 μg/ml) and cultured at 37 °C and 5% CO_2_ for 2–3 weeks.

### Statistical analyses

Statistical analyses were performed using GraphPad Prism 8.2.0 (GraphPad Software, Inc., La Jolla, CA, USA). The data were first analyzed using the Shapiro–Wilk test to evaluate whether they followed a normal distribution. The equality of variance of each group was assessed using the F-test for two-group comparisons and the Brown-Forsythe test for multi-group comparisons.

To compare the two groups for significance, Student’s *t*-test was used when the data sets met the test requirements for distribution and variance. When the data did not follow a normal distribution, a nonparametric Mann–Whitney *U*-test was used. When the data variances were significantly different in the F-test, the Welch’s *t*-test was used.

To compare multiple groups for significance, one-way ANOVA with Tukey’s multiple comparison test was used when the datasets met the test requirements for distribution and variance. When the data did not follow a normal distribution, the nonparametric Kruskal–Wallis test was used. When the data variances were significantly different in the Brown-Forsythe test, Welch’s one-way ANOVA was used. The results are expressed as mean ± standard deviation (SD). *p* < 0.05 was considered statistically significant.

## Supplementary Information

Supplementary Information.

## Data Availability

All data are retained by the corresponding author (T.M) and will be made available from T.M. upon reasonable request.
