# Supplementary material for: Subset of the periodontal ligament expressed leptin receptor contributes to part of hard tissue-forming cells
Source: Sci Rep. 2023 Mar 1;13:3442. doi: 10.1038/s41598-023-30446-w (PMC9977939; doi:10.1038/s41598-023-30446-w)
Supplement: Supplementary file 1 — Supplementary Information. [file 41598_2023_30446_MOESM1_ESM.docx]

**Supplementary Information**

**Subset of the periodontal ligament expressed leptin receptor contributes to part of hard tissue-forming cells**

Hirotsugu Oka ^1#^, Shinichirou Ito ^2#^, Mana Kawakami^3^, Hodaka Sasaki ^1^, Shinichi Abe ^4,5,6^, Satoru Matsunaga ^4,5,6^, Sumiharu Morita ^4^, Taku Noguchi ^4^, Norio Kasahara ^7^, Akihide Tokuyama ^2^, Masataka Kasahara ^2,5,6^, Akira Katakura ^3,5,6^, Yasutomo Yajima ^1,8^, Toshihide Mizoguchi ^5,6*^

^1^Department of Oral and Maxillofacial Implantology, Tokyo Dental College, Tokyo 101-0061, Japan

^2^Department of Pharmacology, Tokyo Dental College, Tokyo 101-0061, Japan

^3^Department of Oral Pathobiological Science and Surgery, Tokyo Dental College, Tokyo 101-0061, Japan

^4^Department of Anatomy, Tokyo Dental College, Tokyo 101-0061, Japan

^5^Oral Health Science Center, Tokyo Dental College, Tokyo 101-0061, Japan

^6^Tokyo Dental College Research Branding Project, Tokyo Dental College, Tokyo 101-0061, Japan

^7^Department of Histology and Developmental Biology, Tokyo Dental College, Tokyo 101-0061, Japan

^8^Matsumoto Dental University, MDU hospital, Implant Center, Nagano 399-0781, Japan

***Corresponding author**

Toshihide Mizoguchi, Ph.D.

Professor, Oral Health Science Center

Tokyo Dental College, Tokyo 101-0061, Japan

Phone: +81-36380 9114

Fax: +81-3-6380 9606

E-mail: tmizoguchi@tdc.ac.jp

#These two authors contributed equally to this work

**
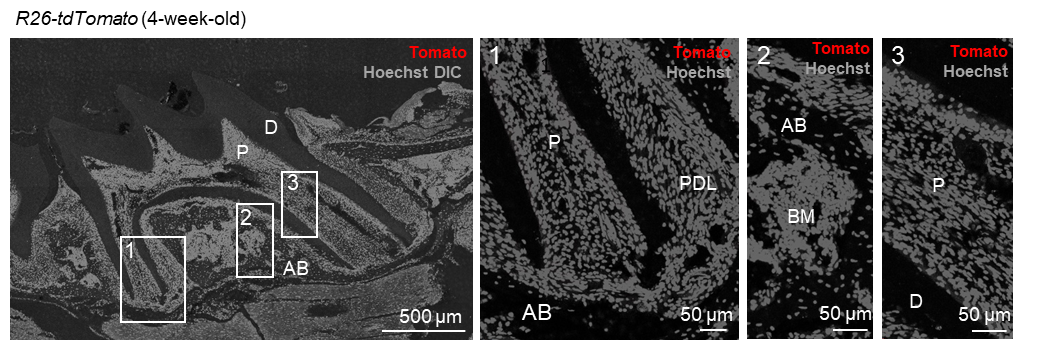
**

**Supplementary Figure S1. Images of maxillary first molar from negative control mouse.**

Representative confocal images (Z stack) of thick maxillary first molar sections from R26-tdTomato control mice; n = 3; scale bar = 500 μm. Numbered panels represent the magnified views of the boxed areas; scale bar = 50 μm. P: pulp, D: dentin, AB: alveolar bone, PDL: periodontal ligament, BM: bone marrow, DIC: differential interference contrast. Nuclei were visualized using Hoechst.


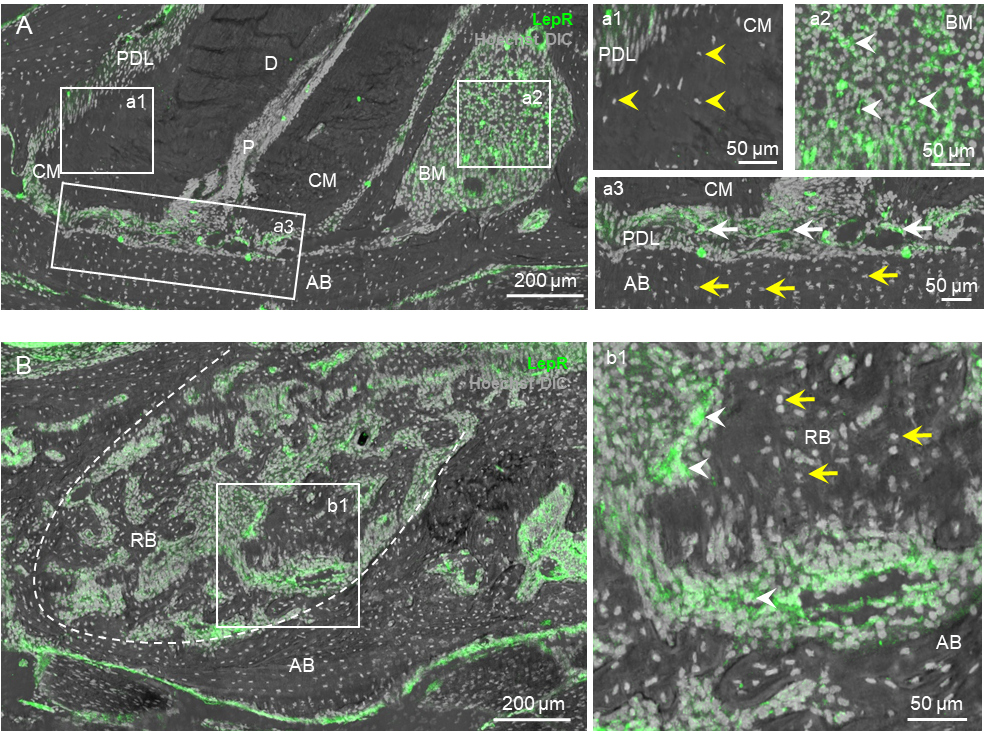


**Supplementary Figure S2. Osteocytes and cementocytes are negative for LepR.**

**A and B.** Representative Z-stack confocal images stained with anti-LepR antibody of thick maxillary first molars of 10-month-old mice (A) and regenerated bone tissue of maxillary first molars 2 weeks post tooth extraction in 4-week-old mice (B); n = 3; scale bar = 200 μm. Numbered panels represent the magnified views of the boxed areas; scale bar = 50 μm. White arrows: LepR^+^ PDL, white arrowheads: LepR^+^ BM stroma, yellow arrows: LepR^−^osteocytes, yellow arrowheads: LepR^–^ cementocytes. D: dentin, AB: alveolar bone, P: pulp, PDL: periodontal ligament, BM: bone marrow, CM: cementum, RB: regenerated bone, DIC: differential interference contrast. Nuclei were visualized using Hoechst.

**
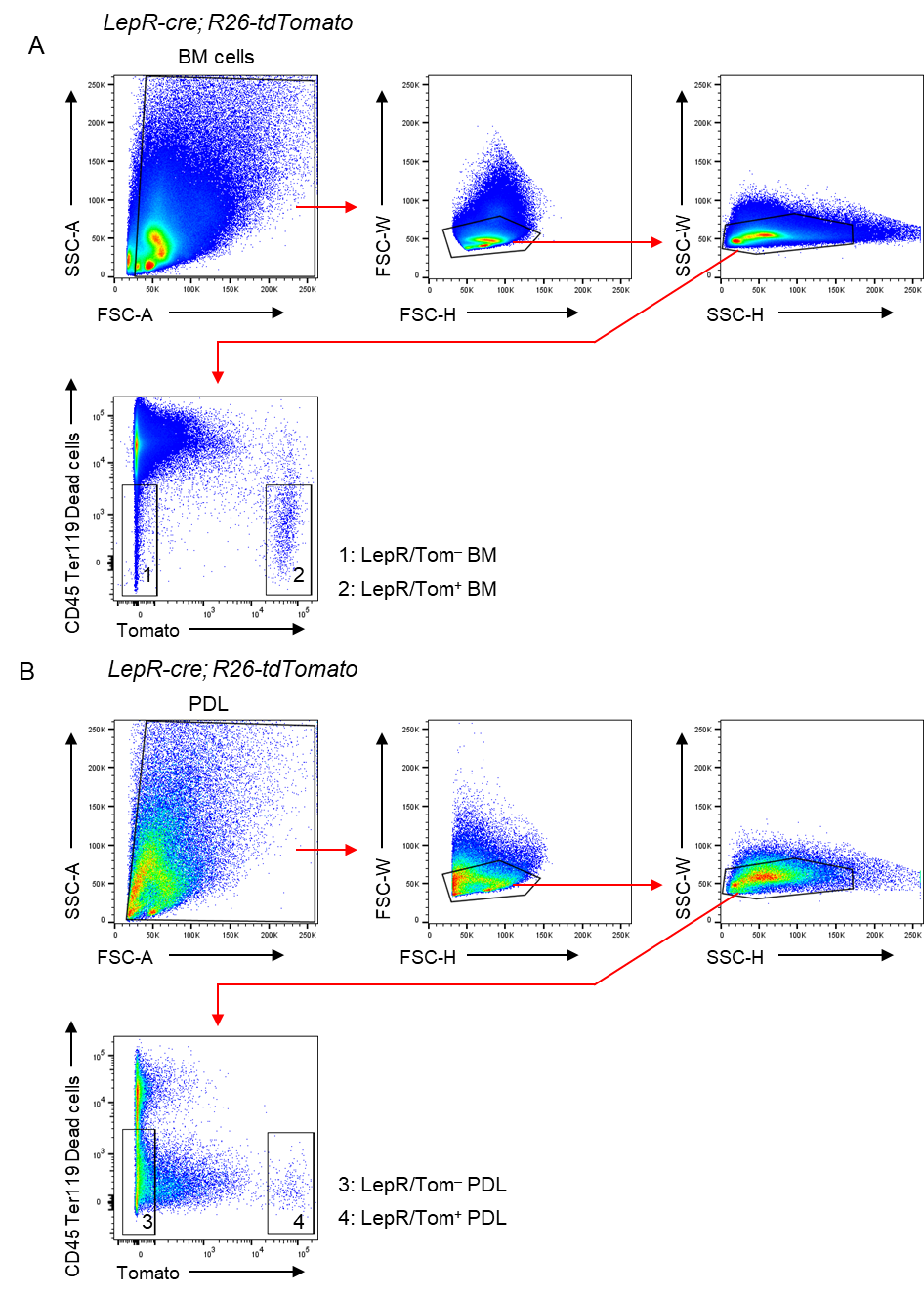
**

**Supplementary Figure S3. Gating strategy used for spheroid formation assay.**

**A and B.** Representative FACS plots showing the gating strategy of flow cytometry for bone marrow (BM) (A) and periodontal ligament (PDL) (B)-derived LepR/Tom^+^ and LepR/Tom^–^ cells.
